# Effects of age and arm movement condition on dynamic balance performance during virtual height exposure

**DOI:** 10.3389/fspor.2025.1705612

**Published:** 2025-12-08

**Authors:** Simon Schedler, Mathew W. Hill, Peter Leinen, Thomas Muehlbauer

**Affiliations:** 1 Division of Movement and Training Sciences/Biomechanics of Sport, University of Duisburg-Essen, Essen, Germany; 2 School of Psychology and Vision Sciences, University of Leicester, Leicester, United Kingdom; 3 Institute of Sport Science, Saarland University, Saarbruecken, Germany

**Keywords:** virtual reality, balance performance, postural threat, arm movement, children, adolescents, young adults (18–29 years)

## Abstract

**Introduction:**

Dynamic balance performance during natural height exposure is affected by age and arm movement condition. However, it remains unclear whether these effects persist during virtual height exposure. The objective of the present study is to investigate the effects of age and arm movement condition during virtual height exposure.

**Methods:**

A sample of 39 children (11.0 ± 0.5 years), 40 adolescents (14.4 ± 0.6 years), and 43 young adults (23.6 ± 3.6 years) performed two balancing trials (one with free and one with restricted arm movements) in a randomized order during virtual height exposure. The time taken to complete the forward and backward traversal of the beam was recorded for analysis. In addition, perceptual outcomes (i.e., instability, task difficulty, fear of falling, and conscious balance processing), presence, and virtual reality (VR) sickness were assessed after each trial.

**Results:**

There were no significant (all *p* > 0.05) effects of arm condition and no arm-by-age group interaction effects. Significant age group effects were observed for the total (*p* < 0.01, Cohen's *d* = 0.48) and the backward balancing time (*p* < 0.01, Cohen's *d* = 0.57), with children performing significantly longer than adolescents. Young adults reported significantly greater task difficulty (*p* < 0.01, Cohen's *d* = 0.59) and stronger involvement (*p* < 0.01, Cohen's *d* = 0.72) compared with adolescents. All variables were also analyzed according to trial number (i.e., first vs. second), revealing that—irrespective of arm condition—all measures significantly (all *p* < 0.05) improved from the first to the second trial. Significant trial-by-age group interactions (all *p* < 0.05, *η*_p_^2^ = 0.06–0.11) indicated that compared to adolescents and young adults, children took longer to cross the beam during the first trial.

**Discussion:**

The present findings suggest that participants quickly habituated to the VR environment, which may have masked potential benefits of free arm movement during dynamic balancing. In contrast, the observed age-related effects differ from those reported in from studies using actual height elevations, highlighting the need for further research on the effects of VR on balance performance and perceptual outcomes.

## Introduction

Free arm movements have been shown to improve balance performance across various age groups [i.e., children (1), adolescents (2), and young/old adults (3)] and across different types of balance tasks [i.e., static (3), dynamic (1), and proactive (2) balance]. From a practical perspective, sufficient dynamic balance performance is especially important during activities of daily life, which usually include movements such as walking or climbing stairs. In this regard, Hill et al. (1) used different tests of dynamic balance performance (i.e., Y-balance test, jump-landing task, and tandem beam walk) to investigate the effects of different arm movement conditions (i.e., free vs. restricted) in healthy children (mean age: 10.6 ± 0.5 years). Restricting arm movements decreased balance performance (except for the jump-landing task), with the largest effect observed for beam walking, where time increased by 1.5 s (≙19.2%) compared to trials with free arm movements. Similarly, Bostrom et al. (4) studied healthy young males (mean age: 24.3 ± 3.0 years) using inverse dynamics analysis of upper-body movements during forward balancing tasks of increasing difficulty (i.e., beam width of 6, 4.5, and 3 cm). They found that upper-body movements become increasingly important as task difficulty increases.

While previous studies demonstrate that arm movements influence balance across ages and tasks, direct comparisons between studies is difficult due to methodological differences (i.e., different tests for different age groups). Muehlbauer et al. (2) addressed this by assessing static (i.e., one-legged stance), dynamic (i.e., beam walking), and proactive (i.e., Y-balance test) balance performance with free vs. restricted arm movements under diverging levels of task difficulty in healthy children (mean age: 11.5 ± 0.6 years), adolescents (mean age: 14.0 ± 1.1 years), and young adults (mean age: 24.7 ± 3.0 years). Restricting arm movements had a greater detrimental effect in children than in young adults, particularly during tasks of high difficulty (e.g., foam ground, small base of support) compared to low difficulty (e.g., firm ground, larger base of support), with the effect being most evident in young individuals. These findings are consistent with a recent systematic review and meta-analysis (5), which reported moderate effects of arm condition on balance performance, especially for difficult tasks.

Mechanistically, it has been argued that free arm movements support balance performance as they enable individuals to produce larger torques to counteract destabilizing body movements (6), aid in the control and shift of the center of mass (COM) away from the direction of instability (7), and increase the distribution of body mass to generate a larger moment of inertia (1). Moreover, age-related differences have been attributed to the still maturing postural control system in children (2).

Beyond task difficulty, arm movements also affect balance under height-induced postural threats, where the consequences of failure are amplified, allowing assessment of both behavioral (e.g., balance performance) and emotional (e.g., perceived stability, fear of falling, or conscious balance processing) responses (8–10). Lambrich et al. (8) examined healthy young adults (mean age 24.4 ± 4.9 years) walking 5 m at ground level (no threat) and on a 0.8-m-high balance beam (threat), under conditions of free or restricted arm movements. Dynamic balance performance (e.g., gait speed, cadence, step time) deteriorated under the threat condition, a situation in part (i.e., step time) amplified when arm movements were restricted. These performance changes were accompanied by reduced perceived stability and by increases in fear of falling and conscious balance processing during postural threat. Wissmann et al. (8) extended this approach to include children (age: 11.1 ± 0.3 years) and young adults (age: 24.0 ± 4.7 years). Under threat (i.e., balancing at 0.8 m), behavioral (i.e., gait speed, cadence), and emotional parameters (i.e., fear of falling, balance confidence, perceived safety, and conscious balance processing) worsened, whereas restricting arm movements only affected gait speed, balance confidence, and fear of falling. Emotional responses were stronger in children than young adults. A significant age group × threat interaction indicated that only young adults reduced their cadence during the threat condition, indicating a potentially insufficient gait strategy (8).

In summary, restricting arm movements and exposing individuals to height-induced postural threat lead to robust changes in dynamic balance performance and emotional state responses when balance tasks are performed in the real visual environment. These changes are further influenced by age, with children usually being more affected by restricting arm movements and postural threat than young adults.

With the growing accessibility of consumer-oriented head-mounted displays (HMDs), virtual reality (VR) is being increasingly employed in postural control research (9–11). A systematic review (12) suggests that VR can be an effective and feasible balance training tool. VR allows participants to experience an almost infinite number of visual environments while physically remaining in the safe environment of a laboratory or gym setting. It also enables manipulation of visual input beyond the usual dichotomic constraints (i.e., eyes open/closed), including the simulation of height-induced postural threat. In the real world, such exposure is typically limited to heights between 0.8 m (13) and 3.2 m (14) due to architectural and safety constraints. While evidence indicates that VR-induced postural threat elicits behavioral and emotional responses comparable to those observed in the real visual environment (11)—at least for static balance (15)—few studies have investigated the combined effect of arm movement condition and age on dynamic balance performance during virtual height exposure. Understanding this interaction is particularly important when VR is employed in the context of balance tests and training.

The aim of the present study was therefore to investigate the effects of arm movement condition (i.e., free vs. restricted) and age group (i.e., children, adolescents, young adults) on dynamic balance performance and emotional state responses when performing a balance task in a virtual environment. Based on findings from real-world postural threat studies (8, 9), we hypothesized performance to be better with free compared to restricted arm movements. We further hypothesized that this effect would be greater in children and adolescents (due to ongoing maturation) as compared to young adults. Finally, we anticipated that changes in performance would be accompanied by changes in emotional responses, including perceived instability, fear of falling, and conscious motor processing.

## Methods

### Participants and sample size calculation

Previous research (8, 15) on the impact of arm movement and postural threat on dynamic balance performance and emotional state outcomes reported moderate to large effects during experiments conducted in the real visual environment. A power analysis conducted with G*Power (16) resulted in a minimum sample size of 54 participants (i.e., 18 per age group) for a 2 (within-subject: free arms vs. restricted arms) × 3 (between-subject: children vs. adolescents vs. young adults) repeated-measures ANOVA with 90% power (standardized medium effect size: *f* = 0.25, *p* = 0.05). As two of the included questionnaires [i.e., conscious balance processing (13) and iGroup Presence Questionnaire (iPQ) (14)] were answered only by distinct subsamples, the total sample size included 39 children (11.0 ± 0.5 years; 49% females), 41 adolescents (14.5 ± 0.7 years; 53% females), and 43 young adults (23.6 ± 3.6 years; 49% females). According to the visual Height Intolerance Severity Scale (vHISS) (17), 2.6% of the children (*N* = 1; vHISS score: 6), 19.5% of the adolescents (*N* = 8; mean vHISS score: 5.5 ± 3.8), and 34% of the young adults (*N* = 15; mean vHISS score: 3.5 ± 2.0) had experienced symptoms of visual height intolerance previously, yet only one adolescent fulfilled the criteria for acrophobia and was therefore excluded. Table 1 provides an overview of participant characteristics. All participants were healthy (i.e., free of any known musculoskeletal or neurological impairments), naïve to the balancing task, and gave their written consent to participate. In addition, written consent was obtained from the legal guardians of minors before the study began. The study was conducted in compliance with the guidelines of the Declaration of Helsinki (1964) and approved by the local Human Ethics Committee.

**Table 1:** Participant characteristics.

Parameter	Children (*N* = 39)		Adolescents (*N* = 40)		Young adults (*N* = 43)	
	Female	Male	Female	Male	Female	Male
*N*	19	20	21	19	21	22
Age (years)	11.1 ± 0.4	10.9 ± 0.6	14.5 ± 0.6	14.5 ± 0.8	23.6 ± 3.9	23.5 ± 3.5
Body height (cm)	149.6 ± 7.5	151.8 ± 8.4	167.0 ± 6.1	**176.5** **± 8.7**	167.0 ± 6.1	**182.1** **± 7.5**
Body mass (kg)	41.2 ± 8.5	46.2 ± 9.0	55.4 ± 8.3	63.8 ± 10.1	64.7 ± 10.1	**86.7** **± 18.1**
Body mass index (kg/m^2^)	18.3 ± 3.0	20.1 ± 3.8	19.8 ± 2.5	20.4 ± 2.2	22.8 ± 3.7	**26.2** **± 5.3**

f, female; and m, male; bold values indicate statistically significant (*p* < 0.05) differences from age group's females.

### Virtual reality scenario

The virtual environment was provided through a consumer-oriented, stereoscopic HMD (Oculus Quest 2, Meta Inc., Menlo Park, USA) using an application (Richie's Plank Experience, Toast VR PTY. LTD., Gold Coast, Australia). In the scenario, participants entered an elevator at ground level, which took them to the 80th floor of a skyscraper, where a virtual wooden beam extended from the elevator. The visual surround involved a virtual city scene with streets, skyscrapers, moving cars, flying helicopters and birds, as well as sounds of traffic and wind. The dimensions of the virtual beam were adjustable; in order to match the beam length of a commonly used beam-walking test [i.e., 3-m beam-walking backward test (18)] as closely as possible, the virtual beam was adjusted to a length of 3 m with the least possible width of 0.1 m.

### Procedures

**Figure 1:**
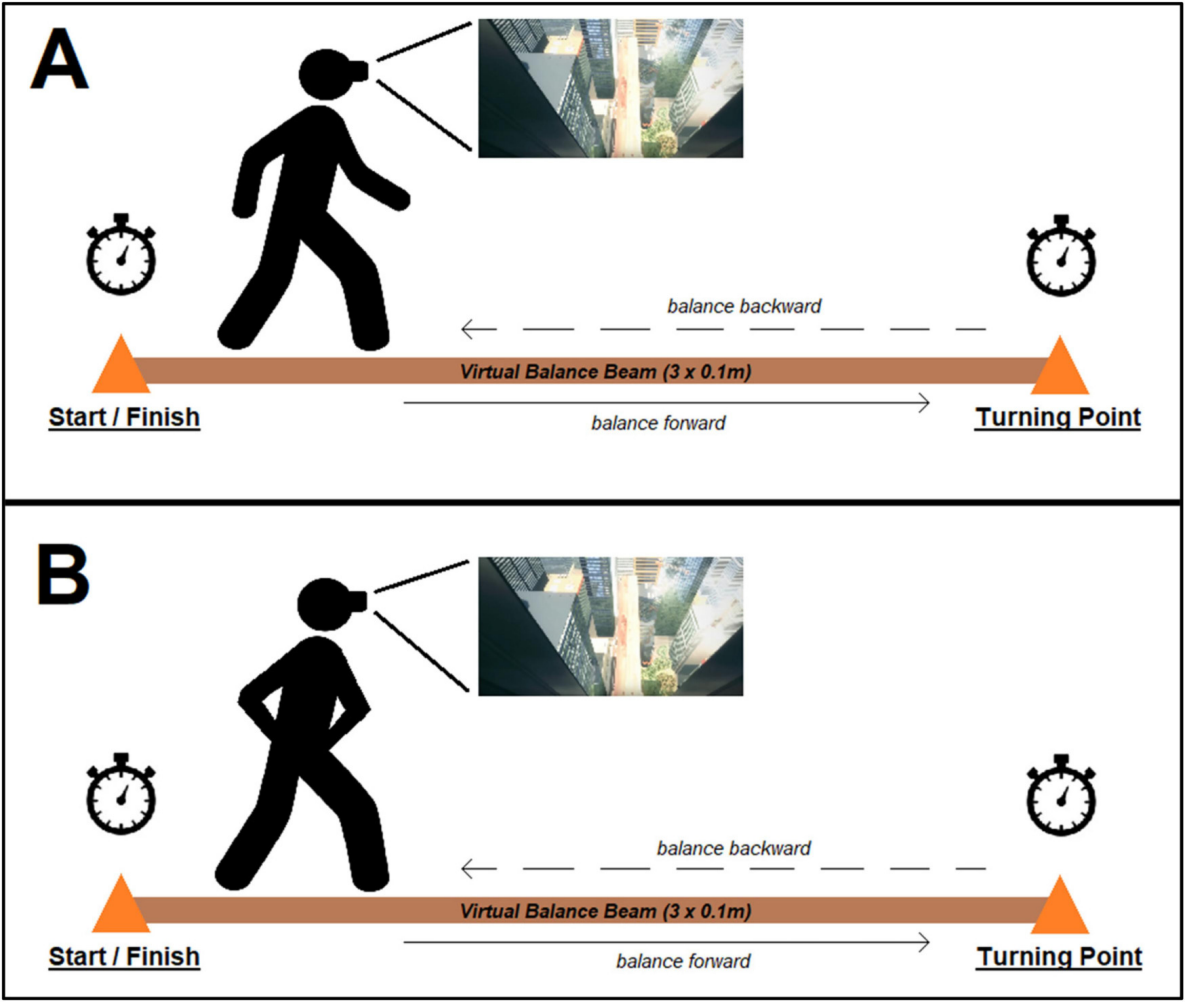
Schematic illustration of the balancing trials with free **(A)** and restricted **(B)** arm movements.

Initially, the body height and mass of participants were assessed using a standardized stadiometer (Seca 217, Basel, Switzerland) and scale (Seca 803, Basel, Switzerland). Before measurements, participants had to answer the vHISS (17) as it has previously been shown that individual height intolerance affects postural and emotional responses when being exposed to virtual heights (9). They were then presented with the HMD (Oculus Quest 2, Meta Inc., USA) and received standardized instructions of what they would see in VR, how they could operate the elevator, and when they should start to balance on the virtual beam. Afterward, they put on the HMD, which was adjusted to their head, and were given a short period of time to become accustomed to the virtual environment (approximately 1 min). Once they confirmed that they felt comfortable with the HMD and the controllers, they were instructed to operate the elevator to the 80th floor and position themselves directly in front of the virtual beam. Subsequently, they received standardized instructions on how to walk on the beam (i.e., with free or restricted arm movement) and were then given the start signal (i.e., “ready—go!”). Afterward, participants balanced forward to the end point of the virtual beam and then returned to the starting point (i.e., elevator) while walking backward. Cones were used to mark the 3-m distance of the virtual beam in the real environment (Figure 1). After completing a trial, participants were instructed to operate the elevator back to ground level. They were then ordered to take off the HMD and had to answer several questionnaires, which took approximately 5 min. Thereafter, they performed a second balancing trial, which followed the same procedure as during the first trial. The only difference between the first and the second trials was whether arm movements were free or restricted. After completing the second trial, participants again had to answer the questionnaire. The sequence of the arm condition was randomized and balanced across all participants. During the “restricted” trials, participants had to keep their hands attached to the anterior superior iliac spine.

Stepping off the beam resulted in an animated fall in the virtual environment and was noted as a failed trial. Participants who experienced a fall were allowed a second trial and reminded “to balance as fast and as safely forth and back across the virtual beam as possible” with particular emphasis on “safely.” In total, we recorded eight falls from seven individuals over the course of the study. The number of failed trials was greater in children (*n* = 5; including one participant with two failed trials) compared to adolescents (*n* = 2) and young adults (*n* = 1). Failed trials were only observed during the first trial, irrespective of arm condition. In both conditions (i.e., arms free and arms restricted), the first successful trial was used for analyses.

### Dynamic balance performance

Dynamic balance performance was assessed by recorded the time needed to complete the beam-walking task in the virtual environment to the nearest 0.01 s using a standardized stopwatch. Moreover, split times for forward and backward balancing were taken.

### Psychometric assessments

#### Visual Height Intolerance Severity Scale

As participants balanced at a significant simulated height (i.e., 80th floor), the German version of the vHISS (17) was utilized to estimate each individual's severity of visual height intolerance on a metric interval scale ranging from 0 to 13. The vHISS consists of 10 questions on the history, occurrence, and symptoms of visual height intolerance. The scale is also suitable for differentiating between acrophobic and non-acrophobic individuals and was answered prior to the start of the measurements.

#### Emotional state outcomes

After each balancing trial, the perceived instability (19), task difficulty (20), and fear of falling (21) of the participants were assessed. Specifically, they were asked to evaluate how stable they felt during the trial, how difficult it was to maintain their balance, and how afraid they were to sustain a fall during the trial. All questions had to be answered on an 11-point visual analog scale (VAS) (i.e., 0 = completely stable/not difficult at all/not afraid at all; 10 = extremely wobbly/extremely difficult/extremely afraid) with higher values indicating greater instability, difficulty, and fear of falling, respectively.

#### Conscious balance processing

A four-item questionnaire (13) was used to estimate each participant's conscious balance processing during balancing trials. Participants were asked to rate whether they (i) consciously thought about keeping their balance, (ii) were aware of the way their body and mind functioned during the task, (iii) were self-conscious about their appearance, and (iv) were concerned about the style of movement during the task on a six-point scale (1 = “strongly disagree”; 6 = “strongly agree”). To grade the level of conscious balance processing, the values were summed up to a total score ranging from 4 to 24, with higher scores indicating a more conscious way of motor processing. The questionnaire was answered after each of the two balance trials.

### Presence

To assess the sense of presence of the participants while being immersed in the virtual environment, they answered the iPQ (14) after each of the two balancing trials. The questionnaire consisted of 14 items measuring the subscales of general presence (i.e., item 1), spatial presence (i.e., items 2–6), involvement (i.e., items 7–10), and experienced realism (i.e., items 11–14) on a seven-point Likert scale (i.e., −3, 3), with smaller values indicating lower perceived presence. To fit the direction of the scale (e.g., smaller values indicating lower presence), the algebraic signs of items 3, 9, and 11 had to be reversed before analysis. In addition, the total score was calculated using the aggregated mean value. All procedures followed the recommendations of Tran et al. (22).

### Virtual reality sickness

To assess virtual reality sickness, participants completed the Virtual Reality Sickness Questionnaire (VRSQ) (23) after each of the two trials. The questionnaire asked whether participants were affected by nine potential side effects (e.g., eyestrain, vertigo) of VR on a four-point scale (i.e., 0 = not affected; 3 = strongly affected). Four symptoms were associated with oculomotor components (e.g., eyestrain), while five symptoms were linked to disorientation (e.g., vertigo). For analysis, an oculomotor score [i.e., ((∑ items 1–4)/12) × 100))], a disorientation score [i.e., (((∑ items 5–9)/15) × 100))], and a total score [i.e., (oculomotor score + disorientation score)/2] were calculated, with higher scores indicating larger virtual reality sickness.

### Statistical analysis

Before conducting the analyses, assumptions of normality and homogeneity of variances/sphericity were checked and met using the Shapiro–Wilk test and Mauchly’s test, respectively. Thereafter, measures of dynamic balance performance, emotional state outcomes, conscious balance processing, presence, and virtual reality sickness were analyzed using a series of repeated-measures ANOVAs to test for the within-subject effects of arm condition [×2 (free vs. restricted)] and the between-subject effects of age group [×3 (children vs. adolescents vs. young adults)]. Based on observations, all parameters were also analyzed with respect to the within-subject effects of trial number [×2 (first vs. second)] and the between-subject effects of age group [×3 (children vs. adolescents vs. young adults)] using repeated-measures ANOVAs. In this regard, “first trial” refers to the first successful trial in condition one (e.g., arms free) and “second trial” refers to the first successful trial in condition two (e.g., arms restricted). If the analyses showed significant effects, Bonferroni-corrected *post hoc* tests (*t*-test) were employed. Using partial eta-squared (*η*_p_^2^), effects of the ANOVA were estimated as being either small (0.02 ≤ *η*_p_^2^ ≤ 0.12), medium (0.13 ≤ *η*_p_^2^ ≤ 0.25), or large (*η*_p_^2^ ≥ 0.26), whereas Cohen's *d* was used to classify the effects of *post hoc* tests as being either trivial (0 ≤ *d* ≤ 0.19), small (0.20 ≤ *d* ≤ 0.49), medium (0.50 ≤ *d* ≤ 0.79), or large (*d* ≥ 0.80). The level of significance was set at *p* ≤ 0.05 and all analyses were performed using JASP version 0.19.3.0 (Amsterdam, The Netherlands).

## Results

### Dynamic balance performance

**Figure 2:**
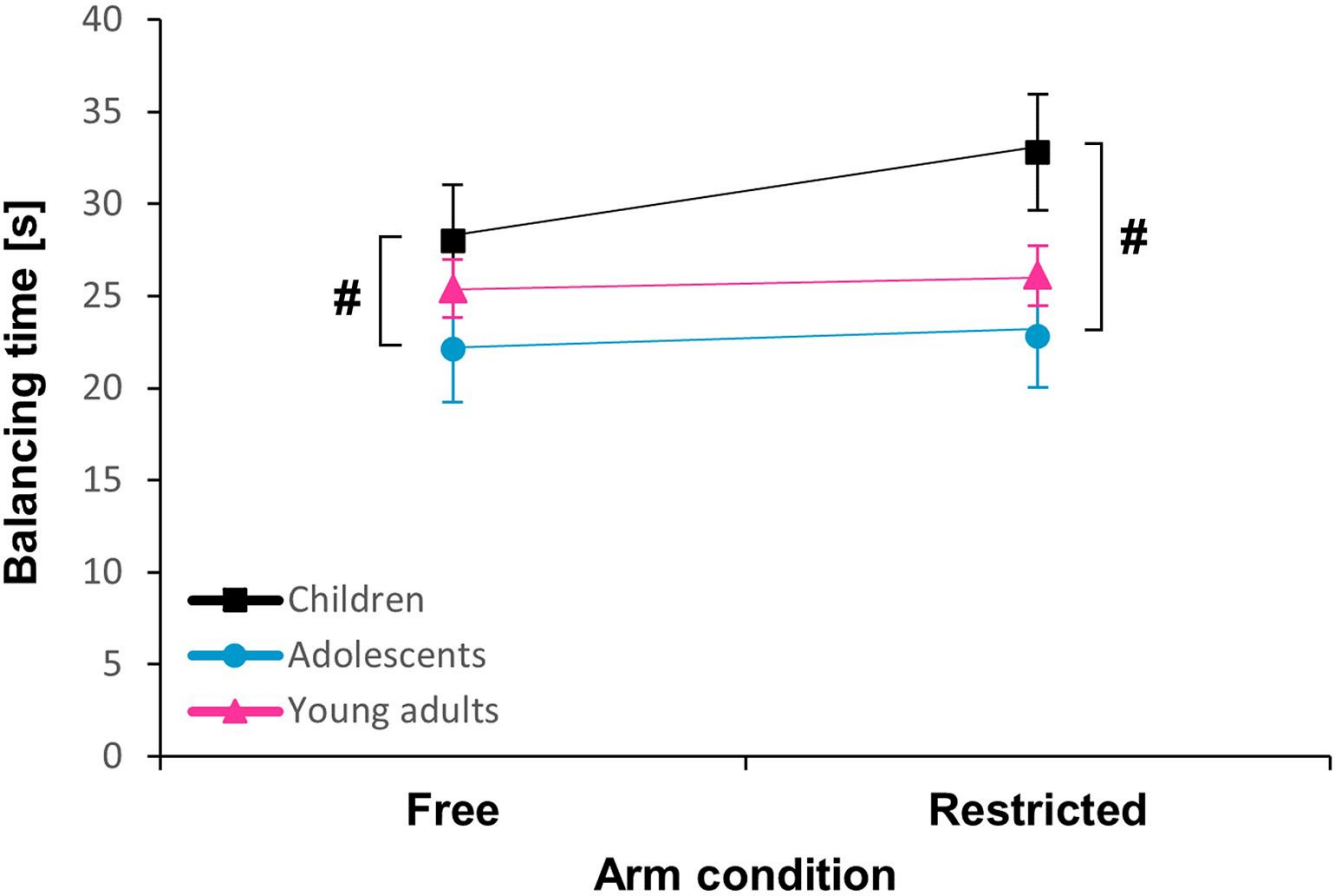
Mean ± SE for balancing time according to arm condition and age group. A hash (#) represents a significant *post hoc* difference between groups (*p* < 0.05).

Descriptive data on dynamic balance performance according to arm condition and age group are presented in Table 2. Analyses revealed no effect of arm condition (*F* = 1.239, *p* > 0.05; *η*_p_^2^ = 0.01) and no arm condition×age group interaction (*F* = 0.554, *p* > 0.05; *η*_p_^2^ = 0.01) (Table 3). However, there was a significant effect of age group (*F* = 3.664, *p* < 0.05; *η*_p_^2^ = 0.06) (Table 3). *Post hoc* tests revealed that children took significantly longer than adolescents to complete the task (*t* = 2.697, *p* < 0.01, Cohen's *d* = 0.48) (Figure 2). For the forward balancing split time, there were no significant effects of arm condition (*F* = 0.802, *p* > 0.05; *η*_p_^2^ = 0.01) or age group (*F* = 1.375, *p* > 0.05; *η*_p_^2^ = 0.02), nor was there any interaction (*F* = 0.286, *p* > 0.05; *η*_p_^2^ = 0.01) between the two factors (Table 3). In terms of the backward balancing split time, there was no significant effect of arm condition (*F* = 1.519, *p* > 0.05; *η*_p_^2^ = 0.01) and no arm condition × age group interaction (*F* = 0.781, *p* > 0.05; *η*_p_^2^ = 0.01) (Table 3). However, there was a significant effect of age group (*F* = 4.863, *p* < 0.05; *η*_p_^2^ = 0.08) (Table 3). *Post hoc* tests showed that children took significantly longer than adolescents during backward balancing (*t* = 3.105, *p* < 0.01, Cohen's *d* = 0.57).

**Table 2:** Mean values ± standard deviations for all dependent variables according to age group and arm condition.

Parameter	Children (*N* = 39)		Adolescents (*N* = 40)		Young adults (*N* = 43)	
	Arms free	Arms restricted	Arms free	Arms restricted	Arms free	Arms restricted
Balancing time (s)						
Forward	12.0 ± 8.3	13.7 ± 8.5	10.6 ± 5.4	10.8 ± 6.2	11.4 ± 8.6	11.8 ± 7.3
Backward	15.9 ± 12.4	19.1 ± 12.5	11.5 ± 5.9	12.0 ± 4.9	14.0 ± 11.0	14.2 ± 11.4
Total	28.0 ± 19.0	32.8 ± 19.8	22.1 ± 9.9	22.8 ± 10.2	25.4 ± 18.9	26.1 ± 18.0
Perceived instability (0–10)	2.6 ± 2.3	3.3 ± 2.5	2.5 ± 2.2	2.6 ± 2.1	3.5 ± 2.4	3.5 ± 2.3
Difficulty (0–10)	2.6 ± 2.0	3.0 ± 2.1	2.2 ± 1.7	2.3 ± 2.0	3.2 ± 2.2	3.4 ± 2.5
Fear of falling (0–10)	2.5 ± 2.4	3.3 ± 2.7	2.6 ± 2.6	2.2 ± 2.3	3.2 ± 2.8	3.1 ± 2.4
Conscious balance processing (4–24)	11.9 ± 3.6	13.4 ± 3.9	15.1 ± 2.5	14.6 ± 3.0	14.1 ± 2.0	14.3 ± 2.7
Presence (−3 to 3)						
Sense of being there	0.52 ± 1.57	0.67 ± 1.16	0.05 ± 1.29	0.14 ± 1.70	0.86 ± 1.28	0.76 ± 1.26
Spatial presence	0.64 ± 1.24	0.63 ± 0.93	0.52 ± 1.04	0.59 ± 1.28	1.05 ± 0.78	0.88 ± 0.88
Involvement	0.37 ± 0.88	0.18 ± 1.03	−0.34 ± 1.10	0.07 ± 1.17	0.67 ± 1.03	0.61 ± 1.19
Experienced realism	−0.18 ± 1.03	−0.17 ± 0.85	−0.47 ± 0.99	−0.44 ± 1.09	−0.25 ± 0.89	−0.25 ± 0.81
Total	0.32 ± 0.91	0.28 ± 0.73	−0.04 ± 0.67	0.11 ± 0.99	0.55 ± 0.68	0.47 ± 0.70
Virtual reality sickness (0–100)						
Oculomotor score	9.0 ± 13.8	11.5 ± 15.5	10.3 ± 12.5	10.4 ± 12.1	13.8 ± 15.2	14.7 ± 17.0
Disorientation score	9.6 ± 18.9	10.6 ± 15.0	9.2 ± 12.1	7.8 ± 11.5	7.1 ± 7.9	9.9 ± 11.8
Total	9.3 ± 15.5	11.1 ± 13.5	9.7 ± 11.2	9.1 ± 10.6	10.5 ± 10.4	12.3 ± 13.3

**Table 3:** Main and interaction effects of the repeated-measures ANOVAs for dynamic balance and psychometric outcomes.

Parameter	Arm (free vs. restricted)		Age group (CH vs. AD vs. YA)		Trial (first vs. second)		Arm × age group interaction		Trial × age group interaction	
	*F*	α (η_p_^2^)	*F*	α (η_p_^2^)	*F*	α (η_p_^2^)	*F*	α (η_p_^2^)	*F*	α (η_p_^2^)
Balancing time (s)										
Forward	0.802	0.372 (<0.01)	1.375	0.257 (0.02)	100.885	**<0.001** **(0.46)**	0.286	0.752 (<0.01)	3.687	**0.028** **(0.06)**
Backward	1.519	0.220 (0.01)	4.863	**<**0**.01** **(**0**.08)**	79.254	**<**0**.001** **(**0**.40)**	0.781	0.460 (0.01)	7.248	**0.001** **(0.11)**
Total	1.239	0.268 (0.01)	3.664	**0.029** **(0.06)**	104.622	**<0.001** **(0.47)**	0.554	0.576 (0.01)	6.605	**0.002** **(0.10)**
Perceived instability (0–10)	1.325	0.252 (0.01)	2.701	0.071 (0.04)	60.355	**<0.001** **(0.34)**	0.573	0.565 (0.01)	0.825	0.441 (0.01)
Difficulty (0–10)	1.792	0.183 (0.02)	3.844	**0.024** **(0.06)**	33.913	**<0.001** **(0.22)**	0.253	0.777 (<0.01)	2.404	0.095 (0.04)
Fear of falling (0–10)	.225	0.636 (<0.01)	1.393	0.252 (0.02)	40.599	**<0.001** **(0.25)**	2.357	0.099 (0.04)	1.489	0.230 (0.02)
Conscious balance processing (4–24)	1.440	0.235 (0.03)	3.103	0.053 (0.10)	7.536	**0.008** **(0.12)**	2.631	0.081 (0.09)	1.819	0.172 (0.06)
Presence (−3 to 3)										
Sense of being there	.104	0.748 (<0.01)	1.833	0.169 (0.01)	1.439	0.235 (0.02)	0.251	0.779 (0.01)	0.721	0.490 (0.02)
Spatial presence	.100	0.753 (<0.01)	1.128	0.330 (0.04)	4.752	**0.033** **(0.07)**	0.396	0.675 (0.01)	0.550	0.580 (0.02)
Involvement	.179	0.674 (<0.01)	3.578	**0.034** **(0.11)**	2.540	0.116 (0.04)	2.139	0.127 (0.07)	0.388	0.680 (0.01)
Experienced realism	.014	0.906 (<0.01)	0.611	0.546 (0.02)	1.548	0.218 (0.02)	0.005	0.995 (<0.01)	1.470	0.238 (0.05)
Total	.012	0.914 (<0.01)	2.369	0.102 (0.07)	5.645	**0.021** **(0.09)**	0.834	0.439 (0.03)	0.313	0.732 (0.01)
Virtual reality sickness (0–100)										
Oculomotor score	1.144	0.287 (0.01)	1.201	0.305 (0.02)	13.172	**<0.001** **(0.10)**	0.447	0.640 (0.01)	0.247	0.782 (<0.01)
Disorientation score	1.016	0.316 (0.01)	0.214	0.808 (<0.01)	5.918	**0.016** **(0.05)**	2.153	0.121 (0.04)	1.074	0.345 (0.02)
Total	1.598	0.209 (0.01)	0.305	0.738 (.01)	13.754	**<0.001** **(0.10)**	0.947	0.391 (0.02)	0.416	0.660 (0.01)

CH, children; AD, adolescents; YA, young adults; bold values indicate statistically significant differences (α < 0.05); 0.02 ≤ η_p_^2^ ≤ 0.12 indicates small, 0.13 ≤ η_p_^2^ ≤ 0.25 indicates medium, and η_p_^2^ ≥ 0.26 indicates large effects.

Table 4 displays descriptive data on dynamic balance performance according to trial number and age group. With regard to total balancing time, the analyses revealed a significant effect of trial (*F* = 104.622, *p* < 0.001; *η*_p_^2^ = 0.47) and a significant trial × age group interaction (*F* = 6.605, *p* < 0.01; *η*_p_^2^ = 0.10) (Table 3). *Post hoc* tests showed that—irrespective of arm movement condition—balancing times during the second trial were significantly shorter than during the first trial (*t* = 10.228, *p* < 0.001, Cohen's *d* = 0.90) and that improvements from the first to the second trial were especially pronounced in children, who took significantly longer than adolescents during the first but not during the second trial. With regard to the forward balancing time, the analyses revealed a significant effect of trial (*F* = 100.885, *p* < 0.001; *η*_p_^2^ = 0.46) and a trial × age group interaction (*F* = 3.687, *p* > 0.05; *η*_p_^2^ = 0.06) (Table 3). *Post hoc* tests again indicated that forward balancing times during the second trial were significantly shorter than during the first trial (*t* = 10.044, *p* < 0.001, Cohen's *d* = 0.93) and that children improved to the level of adolescents and young adults during the second trial. With respect to the backward balancing split time, there was a significant effect of trial number (*F* = 79.254, *p* < 0.001; *η*_p_^2^ = 0.40) and a significant trial × age group interaction (*F* = 7.248, *p* < 0.01; *η*_p_^2^ = 0.11) (Table 3). Backward balancing times during the second trial were significantly shorter than during the first trial (*t* = 8.902, *p* < 0.001, Cohen's *d* = 0.76), and these improvements were again especially evident in children.

**Table 4:** Mean values ± standard deviations for all dependent variables according to age group and trial number.

Parameter	Children (*N* = 39)		Adolescents (*N* = 40)		Young adults (*N* = 43)	
	1st trial	2nd trial	1st trial	2nd trial	1st trial	2nd trial
Balancing time (s)						
Forward	17.2 ± 8.6	**8.6** **± 5.5**	13.0 ± 6.4	**8.4** **± 4.0**	14.4 ± 9.5	**8.9** **± 4.7**
Backward	23.3 ± 12.8	**11.8** **± 9.0**	14.0 ± 5.5	**9.4** **± 4.1**	16.8 ± 13.6	**11.4** **± 7.2**
Total	40.5 ± 19.3	**20.3** **± 13.6**	27.0 ± 10.2	**17.8** **± 7.4**	31.2 ± 22.1	**20.3** **± 11.6**
Perceived instability (0–10)	3.8 ± 2.4	**2.1** **± 2.2**	3.2 ± 2.0	**2.0** **± 2.1**	4.4 ± 2.4	**2.6** **± 1.9**
Difficulty (0–10)	3.4 ± 1.8	**2.2** **± 2.0**	2.5 ± 1.8	**2.0** **± 1.9**	4.0 ± 2.4	**2.7** **± 2.0**
Fear of falling (0–10)	3.7 ± 2.6	**2.0** **± 2.4**	2.8 ± 2.5	**2.0** **± 2.3**	3.9 ± 2.8	**2.4** **± 2.2**
Conscious balance processing (4–24)	13.6 ± 4.2	**11.8** **± 3.2**	15.0 ± 2.3	**14.7** **± 3.2**	14.5 ± 2.0	**13.9** **± 2.7**
Presence (−3 to 3)						
Sense of being there	0.57 ± 1.36	0.62 ± 1.40	0.27 ± 1.42	−0.09 ± 1.57	0.91 ± 1.38	0.71 ± 1.15
Spatial presence	0.79 ± 1.09	**0.48** **± 1.08**	0.72 ± 0.98	**0.39** **± 1.30**	1.00 ± 0.91	**0.92** **± 0.76**
Involvement	0.42 ± 1.01	0.13 ± 0.89	−0.11 ± 1.16	−0.16 ± 1.15	0.77 ± 1.13	0.50 ± 1.08
Experienced realism	−0.02 ± 0.87	−0.32 ± 0.99	−0.50 ± 0.96	−0.41 ± 1.11	−0.18 ± 0.96	−0.32 ± 0.71
Total	0.44 ± 0.80	**0.16** **± 0.82**	0.10 ± 0.78	**−0.03** **± 0.92**	0.59 ± 0.74	**0.43** **± 0.62**
Virtual reality sickness (0–100)						
Oculomotor score	12.6 ± 15.8	**7.9** **± 13.2**	12.4 ± 12.2	**8.6** **± 12.0**	15.7 ± 14.9	**12.8** **± 17.2**
Disorientation score	10.6 ± 15.3	**9.6** **± 18.7**	10.3 ± 12.6	**6.7** **± 10.6**	9.2 ± 11.2	**7.9** **± 8.9**
Total	11.6 ± 13.7	**8.7** **± 15.2**	11.3 ± 11.0	**7.5** **± 10.5**	12.4 ± 11.7	**10.4** **± 12.2**

Bold values indicate statistically significant (*p* < 0.05) differences from age group's first trial.

### Psychometric assessments

For all investigated parameters, no effects of arm condition and no arm × age group interaction effects (all *p* > 0.05) were found. Therefore, the results are presented only with respect to the effect of trial (i.e., first vs. second) and age group (i.e., children vs. adolescents vs. young adults). Descriptive data for psychometric outcomes are displayed according to arm condition (i.e., free, restricted) and trial number (i.e., first, second) in Tables 2 and 4, respectively.

### Emotional state outcomes

#### Perceived instability

**Figure 3:**
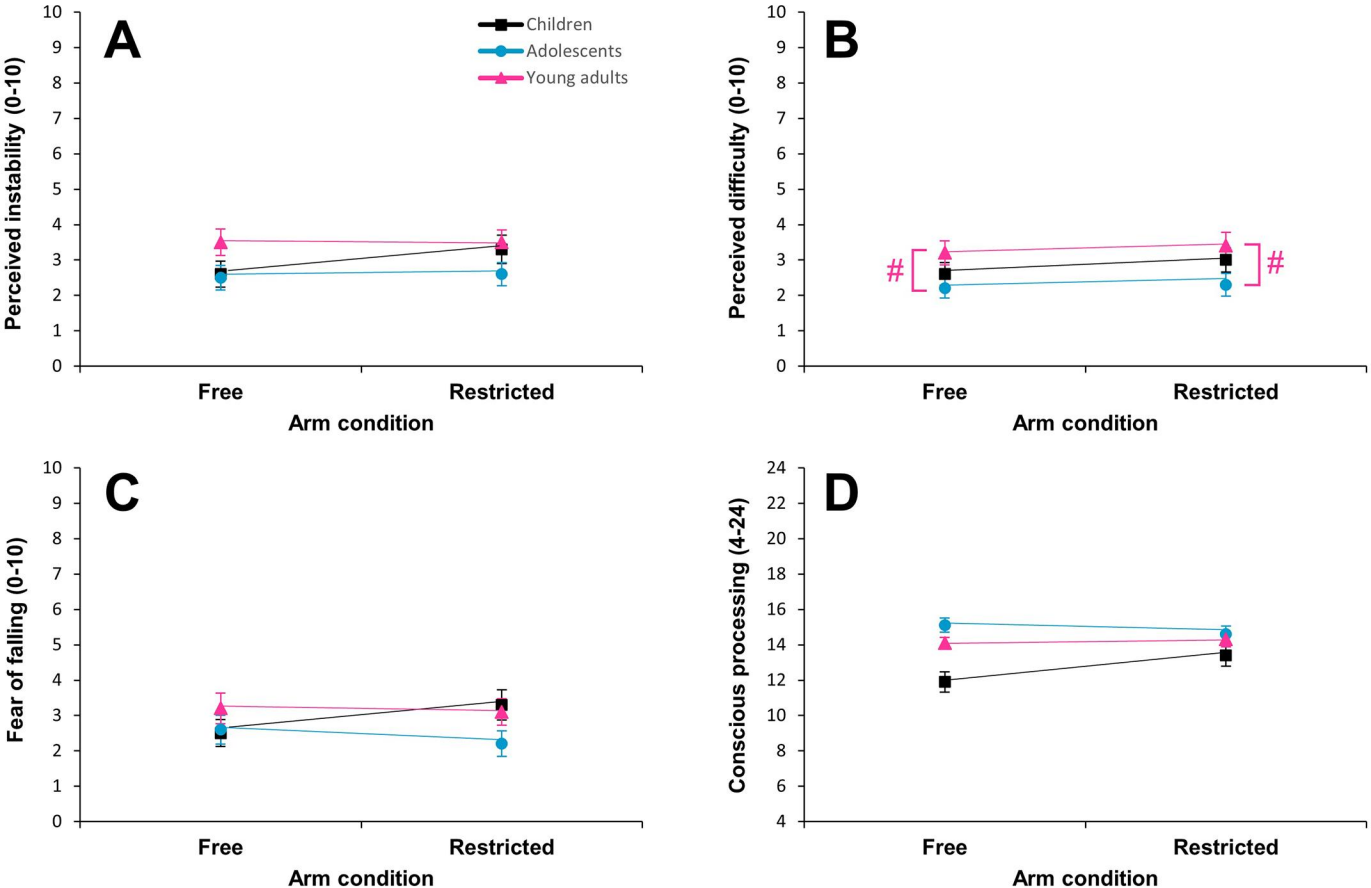
Mean ± SE for perceived instability **(A)**, perceived difficulty **(B)**, fear of falling **(C)**, and conscious balance processing **(D)** according to arm condition and age group. A hash (#) represents a significant *post hoc* difference between groups (*p* < 0.05).

There was a significant effect of trial (*F* = 60.335, *p* < 0.001; *η*_p_^2^ = 0.34), but not of age group (*F* = 2.701, *p* > 0.05; *η*_p_^2^ = 0.04) (Figure 3A) and no trial × age group interaction (*F* = 0.825, *p* > 0.05; *η*_p_^2^ = 0.01) (Table 3). *Post hoc* analyses revealed that perceived instability was significantly greater during the first trial compared to the second (*t* = 7.769, *p* < 0.001, Cohen's *d* = 0.73).

#### Perceived task difficulty

There was a significant effect of trial (*F* = 33.913, *p* < 0.001; *η*_p_^2^ = 0.22) as well as of age group (*F* = 3.844, *p* < 0.05; *η*_p_^2^ = 0.06) (Figure 3B), but no trial × age group interaction (*F* = 2.404, *p* > 0.05; *η*_p_^2^ = 0.04) (Table 3). *Post hoc* analyses revealed that perceived task difficulty was significantly greater during the first trial compared to the second (*t* = 5.824, *p* < 0.001, Cohen's *d* = 0.50) and significantly greater in young adults as compared to adolescents (*t* = −2.772, *p* < 0.05, Cohen's *d* = 0.54).

#### Fear of falling

There was a significant effect of trial (*F* = 40.599, *p* < 0.001; *η*_p_^2^ = 0.25), but not of age group (*F* = 1.393, *p* > 0.05; *η*_p_^2^ = 0.02) (Table 3; Figure 3C). Moreover, there was no trial × age group interaction (*F* = 1.489, *p* > 0.05; *η*_p_^2^ = 0.02) (Table 3). *Post hoc* analyses revealed that fear of falling was significantly greater during the first trial compared to the second in all age groups (*t* = 6.372, *p* < 0.001, Cohen's *d* = 0.55).

### Conscious balance processing

A subsample of 18 children, 18 adolescents, and 22 young adults answered the four-item questionnaire on conscious balance processing (13). Analyses revealed a significant effect of trial (*F* = 7.536, *p* < 0.01; *η*_p_^2^ = 0.12), whereas the effect of age group closely exceeded the level of significance (*F* = 3.103, *p* = 0.053; *η*_p_^2^ = 0.10) (Table 3; Figure 3D). In addition, there was no trial × age group interaction (*F* = 1.819, *p* > 0.05; *η*_p_^2^ = 0.06) (Table 3). *Post hoc* analyses showed that conscious balance processing was significantly larger during the first trial compared to the second (*t* = 2.745, *p* < 0.01, Cohen's *d* = 0.30), especially in children.

### Presence

A subsample of 21 children, 22 adolescents, and 21 young adults answered the iPQ (14). With respect to the total score, there was a significant effect of trial (*F* = 5.645, *p* < 0.05; *η*_p_^2^ = 0.09), but not of age group (*F* = 2.369, *p* > 0.05; *η*_p_^2^ = 0.07), and there was no trial × age group interaction (*F* = 0.313, *p* > 0.05; *η*_p_^2^ = 0.01) (Table 3). *Post hoc* analyses indicated that presence was rated significantly higher after the first trial compared to the second (*t* = 2.376, *p* < 0.05, Cohen's *d* = 0.24). With regard to the subscales of the iPQ, there was a significant effect of trial on spatial presence (*F* = 4.752, *p* < 0.05; *η*_p_^2^ = 0.07) with *post hoc* analyses revealing significantly larger spatial presence after the first trial compared to the second (*t* = 2.180, *p* < 0.05, Cohen's *d* = 0.23) and a significant effect of age on involvement (*F* = 3.578, *p* < 0.05; *η*_p_^2^ = 0.11), which was significantly larger in young adults compared to adolescents (*t* = −2.672, *p* < 0.05, Cohen's *d* = 0.72). No significant effects were found for the subscales “Sense of being there” (all *p* > 0.05) and “Experienced Realism” (all *p* > 0.05).

### Virtual reality sickness

In terms of the total score of the VRSQ, analyses revealed a significant effect of trial (*F* = 13.754, *p* < 0.001; *η*_p_^2^ = 0.10), but not of age group (*F* = 0.305, *p* > 0.05; *η*_p_^2^ < 0.01) (Table 3). Further, there was no significant trial × age group interaction (*F* = 0.416, *p* > 0.05; *η*_p_^2^ < 0.01) (Table 3). *Post hoc* analyses indicated that participants reported significantly larger symptoms of VR sickness after the first trial compared to the second (*t* = 3.709, *p* < 0.01, Cohen's *d* = 0.23). Similar results were obtained for the analyses of the oculomotor and disorientation subscales. More specifically, there was a significant effect of trial (*F* = 13.172, *p* < 0.001; *η*_p_^2^ = 0.10) on oculomotor symptoms of VR sickness, but not of age group (*F* = 1.201, *p* > 0.05; *η*_p_^2^ = 0.02), and there was no trial × age group interaction (*F* = 0.247, *p* > 0.05; *η*_p_^2^ < 0.01) (Table 3). *Post hoc* analyses showed that oculomotor symptoms of VR sickness were significantly greater after the first trial compared to the second (*t* = 3.629, *p* < 0.001, Cohen's *d* = 0.27). Lastly, there was a significant effect of trial (*F* = 5.918, *p* < 0.05; *η*_p_^2^ = 0.05) on the disorientation score, but not of age group (*F* = 0.214, *p* > 0.05; *η*_p_^2^ < 0.01) and no trial × age group interaction (*F* = 1.074, *p* > 0.05; *η*_p_^2^ = 0.02) (Table 3). *Post hoc* analyses showed that disorientation was significantly greater after the first trial compared to the second (*t* = 2.433, *p* < 0.05, Cohen's *d* = 0.15).

## Discussion

The present study investigated the effects of arm movement (i.e., free vs. restricted) and age group (i.e., children, adolescents, and young adults) on proxies of dynamic balance performance, emotional state outcomes, conscious balance processing, presence, and virtual reality sickness in healthy young individuals performing a dynamic balance task during virtual height exposure. Observations made during assessments also motivated analyses examining the effect of trial number (first vs. second). Overall, the results can be summarized as follows: (i) Contrary to our hypotheses, dynamic balance performance was not affected by arm movement condition. (ii) Age group had a significant effect on an individual's dynamic balance performance; however, in contrast to our expectations, children showed worse performances than adolescents, but not in comparison to young adults. (iii) Dynamic balance performance significantly improved from the first trial to the second across all age groups—irrespective of arm movement condition—and these improvements were especially pronounced in children. (iv) Trial-specific changes in dynamic balance performance were accompanied by changes in emotional state outcomes, conscious balance processing, presence, and virtual reality sickness. (v) Age-related differences in psychometric measures were found only for perceived task difficulty and one subscale of the presence questionnaire (i.e., involvement), with young adults scoring significantly higher values than adolescents.

### Effects of arm movement condition and trial order on dynamic balance performance and psychometric outcomes

In the present study, no differences in balancing time were observed between trials with free vs. restricted arm movements. This finding is in contrast to other studies (8, 15), which reported poorer performance during restricted-arm trials compared to free-arm trials in children (8) and young adults (15) exposed to height-induced postural threat. However, the major difference between the present and the previously mentioned studies is that the present study was conducted in a VR environment, whereas the earlier studies were carried out in real-world settings. Although Cleworth et al. (11) found that postural and emotional responses to height-induced postural threat provided through VR were comparable to those observed in real environments, several important differences set their work apart from the current investigation. First, the VR height in their study was relatively modest (3.2 m), whereas the present study simulated a substantially greater threat (>100 m). Second, Cleworth et al. (11) focused on static balance while participants were attached to a safety harness. In the present study, the balance task was more difficult (e.g., walking compared to standing), and participants were free of any safeguard. Consequently, as we could not find differences between performances with free compared to restricted arm movements, it may be speculated that wearing the HMD and/or the unfamiliar visual environment played a major role, potentially masking the typically beneficial role of free arm movements during balance tasks. Supporting this interpretation, a recent systematic review (24) reported decreased dynamic balance performance in young adults wearing an HMD, even when it only displayed a video or a virtual resemblance of the real visual environment.

In support of the previous observations, the effect of trial order showed that there were significant performance increases from the first trial to the second in all investigated age groups, irrespective of arm condition. This pattern may indicate a habituation effect (e.g., decreased responsiveness to the virtual visual stimulus), characterized by significantly shorter balancing times during the second trial. Support for this hypothesis can be derived from studies (25–27) that showed significant improvements in some parameters of standing balance performance during repeated trials under natural height exposure. For example, Zaback et al. (25) investigated static balance performance of 86 healthy young adults (mean age: 22.95 ± 4.06 years) under low (i.e., 0.8 m away from the edge of the support surface) and high (i.e., 3.2 m away from the edge of the support surface) postural threat over five consecutive trials. They observed that some of the initial postural responses to the high threat [e.g., high frequency COP oscillations (>1.8 Hz)] adapted across trials. However, other postural responses persisted over five trials, indicating that on the behavioral level there was no complete habituation to the threat. Despite the large gains from the first trial to the second in our study, participants still took relatively long to traverse the virtual balance beam. Even when only considering the forward gait speed in the faster second trial, children (mean gait speed: 0.35 m/s), adolescents (mean gait speed: 0.36 m/s), and young adults (mean gait speed: 0.33 m/s) remained considerably slower than the 1.0–1.1 m/s reported by Wissmann et al. (8) for children and young adults walking under height-induced postural threat (0.8 m) in a real environment. Consequently, it may be speculated that conducting more trials will foster habituation to the balance task and the unfamiliar visual environment, perhaps to an extent where individuals are able to benefit from free arm movements. However, when designing the study, we anticipated that the effect of the high virtual postural threat would not persist over numerous trials and that the rather simple task of walking 3 m forward and backward on a virtual plank while actually walking on level ground would prove too easy for healthy young individuals to detect an arm condition effect beyond the initial trial. As the results of our study indicate that this is not the case, future studies should investigate the effects of repeated trials in posture-threatening virtual environments conducted with and without arm movements.

Finally, there were large inter-individual differences in individual balancing times even within the age groups. As participants were recruited from local regular schools (i.e., children, adolescents) and the local university (i.e., young adults), variability in dynamic balance performance may have also resulted from different physical activity levels, heterogeneous motor experience, and/or diverse exercise habits. Unfortunately, we did not collect such data as our aim was to analyze the effects of arm condition when performing a dynamic balance task in a posture-threatening virtual environment across different age groups rather than to investigate associations between, for instance, regular physical activity and dynamic balance performance with and without arm movements when balancing in VR. Nevertheless, the high interindividual variability in dynamic balance performance may indicate that factors such as the amount of regular physical activity may play a role and should therefore be investigated in future studies. Moreover, future studies could include a larger number of participants.

In accordance with the findings on balancing times, emotional state outcomes and conscious balance processing did not differ between arm conditions. This contrasts with previous studies (8, 15) conducted under real-world height-induced postural threat, which reported arm condition effects on dynamic balance performance. In the present study, however, there were significant improvements from the first trial to the second. Participants reported greater perceived stability, decreased task difficulty, and reduced fear of falling as well as a shift to a more autonomous balance control, all consistent with considerable habituation to both the task and the VR environment across all age groups. Similar patterns have been reported in studies on static balance performance and emotional responses during natural height exposure (25–27). For instance, Zaback et al. (26) reported that fear of falling decreased significantly over a series of 24 balancing trials at low (i.e., trials 1–2, 23–24; 0.8 m away from the edge of the support surface) and high (i.e., trials 3–22; 3.2 m away from the edge of the support surface) threats in healthy young adults, indicating substantial habituation.

In contrast to balance performance, where values in the present study differed substantially from those reported in real-environment studies of height-induced postural threat, scores for emotional state outcomes and conscious balance processing were relatively comparable. With regard to perceived instability and fear of falling, for instance, Hill et al. (20) reported mean values of 2.9 (young adults) and 4.5 (children) as well as 2.1 (young adults) and 5.0 (children), respectively, during the threat condition (i.e., standing at 0.8 m). The slightly lower values (perceived instability: 2.0–4.4; fear of falling: 2.0–3.9) observed in the present study may indicate that using the high VR-induced postural threat during a dynamic balance task may have exerted stronger effects on behavioral/objective (i.e., balance) measures of healthy young individuals than on the psychometric/subjective (i.e., instability, difficulty, fear of falling, conscious balance processing) data. Future research should examine this potential dissociation more directly.

Finally, the results from the iPQ and the VRSQ questionnaires further support the habituation hypothesis. Both presence and symptoms of virtual reality sickness significantly decreased from the first to the second trial, whereas arm movement condition had no effect. Elevated presence during the first trial may have encouraged a more cautious gait strategy, given the seemingly fatal consequences of task failure such as stepping off and subsequently falling off the beam. By the second trial, participants may have recognized that the VR scenario was fictional and that failures in VR did not translate into real-world consequences. It is worth noting, however, that the observed presence scores were relatively low. Based on iPQ scores across trials, mean values ranged between −0.03 (second try for adolescent') and 0.59 (first try for young adult'), indicating low to moderate presence according to the classification proposed by Tran et al. (22). With respect to symptoms of virtual reality sickness, the observed values were similarly comparably low [range of mean scores: 7.5–12.4 compared to 28.67–42.66 in the study by Kim et al. (23)], and none of the participants withdrew due to VR-sickness-related symptoms. In addition, there was a significant effect of trial on all VRSQ scores, indicating that the light symptoms of VR sickness reported by some participants after the first trial decreased over the two trials, which lends further support to the hypothesis of a habituation effect.

### Effects of age group on dynamic balance performance and psychometric outcomes

Based on previous findings of poorer balance performances in children compared to young adults (28) and evidence of greater visual dependence in children (29), we expected children to perform worse than young adults. However, significant differences were only found between children and adolescents during the first trial, with no differences in performance between children and young adults. This is surprising as the majority of studies report a clear developmental trajectory in balance performance from childhood to young adulthood, typically with substantial differences between children and young adults (28, 30). One possible explanation is that, at the behavioral level, children may be more affected by the HMD, the unfamiliar visual environment, and/or the high virtual postural threat during the first trial than the other two age groups. Notably, improvements from the first trial to the second were greater in children (≙49%) than in adolescents (≙34%) and young adults (≙35%). By the second trial, children demonstrated performances comparable to those of adolescents and young adults, suggesting a slower but ultimately sufficient postural control adaptation.

Nevertheless, we did not find superior balance performance in young adults, who were also outperformed by adolescents, although the difference was not statistically significant. One possible explanation for this finding may relate to the use of VR in the present study. Regular gaming is reportedly most prevalent among adolescents, whereas children and young adults spend comparatively less time playing video games (31). Therefore, it may be speculated that adolescents may have been less affected by the virtual environment than participants from the other two age groups. Although we attempted to control for prior VR experience by including only novices, this characteristic was assessed via self-report and we did not assess each participant's general gaming habits. Consequently, we cannot completely rule out that some participants may nonetheless have had prior experience with VR, which may have been especially likely in the gaming-affine age group of adolescents.

With respect to emotional state outcomes, young adults reported significantly higher task difficulty than adolescents, although there were no age-related differences relating to perceived instability and fear of falling. Thus, while children were more affected at the behavioral level (i.e., dynamic balance performance) during the first trial, without corresponding differences in emotional outcomes (e.g., perceived task difficulty), young adults appeared more affected at the emotional level (i.e., perceived task difficulty), without corresponding differences in behavior (i.e., dynamic balance performance). Interestingly, this pattern contrasts with the findings of Wissmann and colleagues (8), who reported significant effects of postural threat and arm condition on children's emotional responses but not on the behavioral performance (i.e., dynamic balance), whereas the reverse effects was noted in young adults. The reason for this inconsistency remains unclear and warrants further investigation. Potential factors include the nature of the postural threat (i.e., natural height vs. virtual height) as well as its magnitude (i.e., 0.8 m vs. approximately >100 m).

In terms of conscious balance processing, results indicated a trend toward a more automatic method of balance control during virtual height exposure in children as compared to adolescents and young adults, although the effect of age narrowly missed statistical significance (*p* = 0.053, *η*_p_^2^ = 0.10). This finding is again in sharp contrast to the effects of natural height exposure, where automatic balance control is reportedly significantly greater in young adults as compared to children (8). Normally, a more automatic mode of balance processing is associated with better balance performance. However, in the present study, we found the opposite as children showed poorer balance performance than adolescents, at least during the first trial. This discrepancy is another indicator that with respect to the factor age, virtual height exposure may have distinctly different effects on the behavioral (i.e., dynamic balance performance) and emotional (i.e., task difficulty, conscious balance processing) responses of healthy young individuals. Except for the iPQ subscale “Involvement,” which was significantly greater in young adults as compared to adolescents, there were no age-related differences regarding an individual's perceived presence in the virtual environment. In addition, there were no age-related differences in the occurrence of potentially adverse effects of VR between children, adolescents, and young adults. Therefore, the applied technology and virtual setting appear feasible for healthy young individuals, including minors as young as 11 years, at least when it is used over a short time period (approximately 2–10 min).

To summarize, compared to adolescents, children were especially affected on the behavioral level (i.e., balance performance), at least during the first trial, whereas there were no differences between emotional state outcomes among children and adolescent'. On the contrary, young adults were especially affected on the emotional level (i.e., perceived task difficulty), which however did not affect their performance on the behavioral level (i.e., dynamic balance performance), when compared to adolescents.

## Limitations

There are a few limitations of the present study, which need to be addressed. First, our aim was to gain insights into the effects of arm movement condition on dynamic balance performance during virtual height exposure in the general population. Therefore, participants were recruited from local schools (i.e., children, adolescents) and the local university (i.e., young adults). A potential limitation of this approach is that the study groups were most likely rather heterogeneous with respect to their level of physical activity, motor experience, exercise habits, and gaming behavior. As we did not control for these measures, they may have contributed to the partially high variability. Future studies should focus on associations between factors such as exercise habits or gaming behavior and the effects of arm movement condition during virtual height exposure, for instance, by comparing extreme groups (e.g., gymnasts vs. swimmers; intensive video gamers vs. non-gamers).

In addition, our sample was rather heterogeneous with respect to anthropometric characteristics. Future studies, could more closely investigate potential relationships between factors such as foot length or body composition and dynamic balance performance during virtual height exposure.

Another limitation of this study is the use of an incongruent support surface. More specifically, participants balanced across a wooden beam in the virtual environment, whereas in reality they walked on plain ground, which may have produced an intersensory conflict as visual input differed from somatosensory input. However, following pilot trials, we concluded that the balancing task would be too difficult when using a congruent support surface (e.g., low-height wooden beam), especially with respect to the younger individuals (e.g., children). Nevertheless, the absence of proper somatosensory feedback may have affected the outcomes of our study and the balance strategies of the individuals. Future studies could therefore investigate the effects of arm movement condition on dynamic balance performance during virtual height exposure—at least in adolescents and/or young adults—by using a low-height physical beam (e.g., 2–3 cm high) to improve the ecological validity.

Finally, as our study design included only two balancing trials during virtual height exposure, we could not further investigate the observed habituation effect. Future studies should therefore include a larger number of trials (e.g., 10+) and also focus on the vestibular component (e.g., using stochastic vestibular stimulation) of habituation as both of these approaches have been used effectively during natural height exposure in previous studies (26, 27, 32, 33).

## Conclusion

To the best of our knowledge, this study is the first to investigate the effects of arm movement and age group on dynamic balance performance during virtual height exposure. Unlike studies conducted in the real visual environment, there was no effect of arm movement condition when only two trials were performed. However, substantial improvements in both behavioral and emotional outcomes from the first trial to the second indicate a habituation effect to the virtual environment across all age groups. Compared to adolescents, balancing in a virtual environment of high postural threat had a greater effect on the dynamic balance performance of children and a stronger impact on emotional state outcomes of young adults. This pattern is contrary to results from studies using natural height exposure. A potential explanation for adolescents being less affected by VR than children and young adults may be that the former perhaps spend more time playing video games, although this hypothesis needs to be proven in future studies. Overall, results from this study suggest that VR has distinct effects on healthy young individuals' dynamic balance performance and emotional outcomes, which are at least in part different from those reported in studies conducted in the real visual environment. Future studies should therefore investigate the effect of a large number of repeated trials, different visual environments, practical experience with VR, regular gaming, and exercise habits on the balance performance of individuals when exposed to VR.

## Data Availability

The raw data supporting the conclusions of this article will be made available by the authors, without undue reservation.
